# The Significance of YKL-40, a Marker Involved in Extracellular Matrix Remodeling, in Preterm Prelabor Rupture of Membranes: A Prospective Observational Study

**DOI:** 10.3390/diagnostics16172845

**Published:** 2026-09-04

**Authors:** Gülten Çirkin Tekeş, Dinçer Sümer, Gunel Aliyeva, Özge Öztürk, Figen Günday, Nurten Çilek, Zeynep Tuğçe Aşan Özbek, Kadriye Yakut Yücel

**Affiliations:** Perinatology Department, Etlik City Hospital, Ministry of Health, Varlık Mah. Halil Sezai Erkut Cad., Yenimahalle, Ankara 06170, Turkey; drdincersumer@gmail.com (D.S.); dr.aliyeva87@gmail.com (G.A.);

**Keywords:** preterm prelabor rupture of membranes, YKL-40, CHI3L1, composite adverse neonatal outcome, extracellular matrix remodeling, inflammation

## Abstract

**Objective**: Our objectives were to evaluate maternal serum YKL-40 levels in pregnancies complicated by preterm prelabor rupture of membranes (PPROM), compare them with healthy gestational age-comparable controls, and assess the association between YKL-40 levels and subsequent composite adverse neonatal outcomes (CANOs). **Methods**: This prospective observational study included 44 women with PPROM and 44 healthy pregnant controls between 24 and 37 weeks of gestation. Maternal serum YKL-40 concentrations were measured using an enzyme-linked immunosorbent assay (ELISA), with concentrations corrected for the manufacturer-specified 5-fold sample dilution. Multivariable Firth’s penalized logistic regression was used to evaluate the independent association of YKL-40 with PPROM and, within the PPROM cohort only, subsequent CANO. Exploratory receiver operating characteristic (ROC) analysis was performed to assess the ability of YKL-40 to discriminate established PPROM cases from healthy controls. The study was powered a priori only for the primary between-group comparison of YKL-40; the ROC and CANO analyses were not separately powered and are reported as exploratory. **Results**: Maternal serum YKL-40 levels were significantly higher in the PPROM group than in the control group (5.87 ± 2.64 vs. 2.44 ± 0.96 ng/mL, *p* < 0.001). In multivariable analysis, YKL-40 remained independently associated with PPROM (adjusted odds ratio 5.61, 95% CI 2.07–15.23, *p* < 0.001 per 1 ng/mL increase). Within the PPROM cohort, YKL-40 was not independently associated with CANO (*p* = 0.775). Exploratory ROC analysis yielded an AUC of 0.935 (95% CI 0.881–0.989); a ROC-derived cut-off of >3.3 ng/mL provided 86.4% sensitivity and 84.1% specificity for discriminating established PPROM cases from healthy controls. **Conclusions**: Maternal serum YKL-40 levels were significantly elevated in women with established PPROM and remained independently associated with PPROM status, but were not associated with subsequent CANO. Because YKL-40 was measured after PPROM diagnosis, the observed ROC performance reflects case–control discrimination rather than prospective prediction or established diagnostic utility. Larger prospective studies using clinically relevant comparison groups and independently validated assays are required to determine the potential clinical value of YKL-40 in PPROM.

## 1. Introduction

Preterm prelabor rupture of membranes (PPROM) affects approximately 3–4% of pregnancies and complicates about half of all preterm births [1,2]. Despite advances in neonatal care, PPROM remains a major cause of neonatal morbidity and mortality [3,4]. It is associated with adverse perinatal outcomes, including neonatal intensive care unit admission, neonatal sepsis, chorioamnionitis, fetal distress, and complications related to prematurity. Although its etiopathogenesis remains incompletely understood, inflammatory activation, oxidative stress, extracellular matrix degradation, and matrix metalloproteinase activity are considered important contributors to fetal membrane weakening and rupture.

Inflammation is thought to play a central role in both the pathophysiology of PPROM and the development of adverse perinatal outcomes [5,6,7]. Several inflammatory biomarkers, including soluble urokinase-type plasminogen activator receptor, interleukin-6, Parkinson’s disease protein 7, and syndecan-1, have been investigated in relation to PPROM and its perinatal consequences [7,8,9]. In addition to inflammatory mediators, first-trimester circulating biochemical markers have been examined: lower first-trimester pregnancy-associated plasma protein-A (PAPP-A) and free β-human chorionic gonadotropin concentrations have both been associated with the subsequent risk of pPROM and spontaneous preterm birth, although their discriminatory performance appears insufficient for individual-level clinical prediction [10]. However, no circulating biomarker has yet demonstrated sufficient and consistently validated clinical utility in this setting.

YKL-40 (chitinase-3-like protein 1, CHI3L1) is a glycoprotein that binds chitin and heparin but lacks enzymatic chitinase activity [11,12,13]. It is secreted by several cell types involved in inflammatory and tissue-remodeling processes, including macrophages, neutrophils, endothelial cells, and fibroblast-like cells [14]. Through receptor-mediated intracellular signaling pathways, YKL-40 has been implicated in inflammation, apoptosis, oxidative stress responses, angiogenesis, and extracellular matrix (ECM) remodeling [15,16]. Accordingly, circulating YKL-40 has been investigated as a biomarker in several chronic inflammatory and fibrotic disorders. In pregnancy, YKL-40 has been evaluated in conditions such as preeclampsia, small-for-gestational-age pregnancies, intrahepatic cholestasis of pregnancy, and gestational diabetes mellitus; however, its association with PPROM has not previously been investigated.

The biomechanical integrity of the fetal membranes depends on tightly regulated extracellular matrix homeostasis. In PPROM, disruption of this balance, together with inflammation-driven proteolytic activity, contributes to collagen degradation and progressive weakening of the amnion and chorion. Most biomarkers previously studied in PPROM predominantly reflect systemic or local inflammatory activity, whereas YKL-40 is of particular interest because it is associated with both inflammatory signaling and extracellular matrix remodeling. This dual biological role provides a plausible rationale for investigating whether circulating YKL-40 differs in pregnancies with established PPROM.

Therefore, the present study aimed to compare maternal serum YKL-40 concentrations between women with PPROM and gestational age-comparable healthy pregnant controls and to evaluate the association between YKL-40 levels and subsequent adverse perinatal outcomes within the PPROM cohort. Because blood sampling was performed after the diagnosis of PPROM, the between-group analysis was intended to evaluate the association and discriminatory characteristics of YKL-40 in established disease rather than its ability to predict the future development of PPROM.

## 2. Materials and Methods

### 2.1. Study Design and Patient Selection

This prospective observational study was conducted at the Department of Perinatology, Etlik City Hospital, Ankara, Türkiye, between September 2025 and March 2026. The study protocol was approved by the Etlik City Hospital Clinical Research Ethics Committee (approval number: AEŞH-BADEK2-2025-297; approval date: 5 August 2025), and the study was conducted in accordance with the Declaration of Helsinki. Written informed consent was obtained from all participants before enrollment.

Participant recruitment, clinical assessment, and maternal blood sampling were performed prospectively. Neonatal outcomes were subsequently ascertained after delivery through review of standardized hospital neonatal records. The study included two analytically distinct components. The comparison of maternal serum YKL-40 levels between women with established PPROM and healthy controls was cross-sectional with respect to PPROM status, because blood samples were obtained after the diagnosis of membrane rupture. Accordingly, this analysis was intended to assess the association and discriminatory characteristics of YKL-40 in established PPROM rather than its ability to predict the future development of PPROM. In contrast, the analysis evaluating the relationship between YKL-40 levels measured at enrollment and subsequent adverse perinatal outcomes within the PPROM cohort was prospective in nature. No further comparison arms were recruited. In particular, women presenting with clinically suspected but subsequently unconfirmed membrane rupture, women in preterm labour with intact membranes, and women delivering preterm for other indications were not enrolled. The design therefore permits evaluation of the association between maternal serum YKL-40 and established PPROM, but not of the ability of YKL-40 to distinguish PPROM from the conditions with which it is clinically confused.

Eligible women with PPROM were consecutively enrolled at their initial presentation during the study period. Healthy controls were recruited contemporaneously from pregnant women attending routine antenatal outpatient visits at the same institution. Controls were recruited by prospective frequency balancing on gestational age at blood sampling: as PPROM cases were enrolled, control recruitment was directed towards the corresponding gestational weeks, so that the distribution of sampling weeks in the control group approximated that of the PPROM group, and control blood samples were obtained at comparable gestational ages. No individual (1:1) matching was performed, no formal statistical matching algorithm was applied, and the two groups were not treated as matched pairs. Maternal age, gravidity, parity and booking body mass index (BMI) were not actively balanced; the comparability observed for these characteristics (Table 1) reflects the source population rather than the recruitment procedure. The number of eligible women approached who declined participation was not recorded prospectively. Accordingly, between-group comparisons and multivariable analyses were performed using independent-sample methods. The gestational age range of 24–37 weeks refers to the timing of blood sampling and not to the timing of delivery. Controls were followed until delivery, and their pregnancy outcomes were recorded; all but one control delivered at term (mean gestational age at delivery 38.67 ± 1.06 weeks).

All participants were between 24 and 37 weeks of gestation. Exclusion criteria were multiple pregnancy; fetal anomalies identified antenatally at the initial assessment or during routine obstetric follow-up; maternal liver, renal, cardiac, hematological, thyroid, autoimmune, or malignant disease; previous chemotherapy or radiotherapy; intrauterine fetal death; pregnancy achieved through assisted reproductive techniques; smoking, alcohol, or substance use; and clinical or laboratory evidence of an acute or chronic inflammatory condition unrelated to pregnancy or to PPROM itself. This last criterion was restricted to active infection outside the genital tract (for example, respiratory or urinary tract infection under treatment), and to autoimmune, rheumatological or other chronic inflammatory disease. Elevated inflammatory markers attributable to PPROM were not treated as an exclusion criterion, and women who subsequently developed clinical chorioamnionitis were retained in the cohort. Two women with PPROM were excluded on this basis, both because of a documented extragenital infection requiring treatment at presentation. Pregnancies complicated by preeclampsia, fetal growth restriction, or gestational diabetes mellitus were also excluded. Participants with incomplete clinical data or who delivered at another institution were excluded from the final analysis. Fetal anomalies diagnosed only after birth were not considered exclusion criteria because this information was not available at the time of enrollment.

### 2.2. Sample Size Calculation

The sample size was calculated using G*Power version 3.1.9.7 for the primary comparison of maternal serum YKL-40 levels between the PPROM and control groups. In the absence of previous studies or pilot data evaluating maternal YKL-40 levels in PPROM, a large standardized effect size (Cohen’s d = 0.70) was assumed a priori. Using a two-tailed independent-samples *t*-test, with an alpha level of 0.05, 90% statistical power, and a 1:1 allocation ratio, the minimum required sample size was calculated as 44 participants per group (88 participants in total). The study was therefore powered for the primary between-group comparison of YKL-40 levels; ROC and multivariable regression analyses were considered secondary exploratory analyses and were not independently used for sample size determination. No a priori sample size calculation was performed for diagnostic discrimination or for the prediction of neonatal outcomes, and the study was not powered for these analyses. In particular, the CANO analysis was limited by a small number of non-events, which constrains the number of covariates that a multivariable model can support and increases the risk of a type II error. Future studies addressing the diagnostic performance of YKL-40 should be sized a priori for a target area under the curve, and studies addressing neonatal outcomes should be sized on the number of outcome events rather than on the number of participants. No target events-per-variable ratio was specified in advance for the multivariable analyses either. Applying the conventional requirement of at least ten observations per candidate covariate in the smaller outcome category, and taking the 13 neonates without the composite outcome as the limiting quantity, a two-covariate model would require approximately 20 and a three-covariate model approximately 30 such neonates; the present cohort therefore supports only a deliberately restricted, exploratory model. Future work should accordingly derive three separate a priori sample sizes: one for the comparison of biomarker concentrations between groups, one for diagnostic discrimination against a clinically relevant comparator group, sized on a target area under the curve with a prespecified confidence interval width, and one for the multivariable prediction of neonatal outcome, sized on the number of events in the smaller outcome category so that an adequate events-per-variable ratio is achieved.

### 2.3. Diagnosis and Clinical Management of PPROM

The diagnosis of PPROM was established through the direct visualization of amniotic fluid pooling in the posterior vaginal fornix during sterile speculum examination or by a positive biochemical test for membrane rupture [AmniSure (placental alpha-microglobulin-1) and/or Actim PROM (insulin-like growth factor-binding protein-1)]. Following confirmation of PPROM, patients underwent digital vaginal examination when clinically indicated, ultrasonographic assessment, non-stress testing (NST), and laboratory evaluation.

All PPROM cases were managed according to an institutional protocol aligned with ACOG recommendations and tailored to gestational age and maternal–fetal clinical status. Patients at <34 weeks of gestation without indications for immediate delivery, such as clinical chorioamnionitis, placental abruption, non-reassuring fetal status, or active labor, were hospitalized and managed expectantly with close maternal and fetal surveillance. When indicated between 24 + 0 and 33 + 6 weeks of gestation, a single course of antenatal corticosteroids was administered for fetal lung maturation (betamethasone 12 mg intramuscularly, two doses 24 h apart). The latency antibiotic regimen consisted of intravenous ampicillin 2 g every 6 h for 48 h, followed by oral amoxicillin 250 mg every 8 h for 5 days. Patients were closely monitored for clinical chorioamnionitis, fetal compromise, placental abruption, and preterm labor, and delivery was undertaken when maternal or fetal indications for pregnancy termination developed. For PPROM occurring between 34 + 0 and 36 + 6 weeks of gestation, delivery was generally recommended rather than continued expectant management, in accordance with the institutional protocol, although brief individualized observation was permitted when clinically appropriate. Because of this protocol, delivery at 34 + 0 weeks was the single most frequent outcome in the cohort, and gestational age at delivery was therefore partly determined by management policy rather than by disease severity alone.

### 2.4. Definitions of Terms

Maternal clinical and laboratory variables were collected prospectively at enrollment and during inpatient follow-up. Neonatal outcomes were ascertained after delivery through retrospective review of hospital electronic medical records and standardized neonatal charts. The full definition of every component of the composite outcome is provided in Supplementary Table S1.

Neonatal metabolic acidosis was identified based on neonatal clinical and laboratory assessment as documented in the neonatal medical records (arterial pH < 7.20 and/or base deficit > 10 mmol/L or bicarbonate < 15 mmol/L). Need for resuscitation was defined as delivery-room resuscitation beyond routine care, comprising positive-pressure ventilation, intubation, chest compressions or the administration of adrenaline, as recorded by the attending neonatology team. Respiratory distress syndrome (RDS) was diagnosed based on clinical respiratory distress accompanied by compatible radiological findings. Prolonged oxygen support was defined as the requirement for supplemental oxygen extending beyond immediate delivery-room resuscitation, as documented by the attending neonatology team. Neonatal sepsis was diagnosed based on clinical assessment and supporting laboratory findings. Intraventricular hemorrhage (IVH), necrotizing enterocolitis (NEC), pulmonary hypoplasia, and early neonatal death were identified from standardized neonatal records; NEC and RDS were recorded as binary outcomes without severity staging.

Composite adverse neonatal outcome (CANO) was defined a priori as the occurrence of at least one of the following neonatal complications during the early postnatal period: metabolic acidosis, need for resuscitation as defined above, RDS, surfactant requirement, mechanical ventilation and/or prolonged oxygen support, inotropic support, neonatal sepsis, IVH, NEC, pulmonary hypoplasia, or early neonatal death. Admission to the neonatal intensive care unit was not itself a component of CANO, and neonates admitted for observation of prematurity alone, without any of the components listed above, were classified as not having CANO. NICU admission is reported separately as a descriptive outcome.

CANO was analyzed as an unweighted binary composite, with the occurrence of at least one component considered sufficient to meet the endpoint. Outcome data were extracted using predefined criteria by investigators blinded to maternal YKL-40 results.

### 2.5. Laboratory Measurements and YKL-40 Assay

Maternal blood parameters, including hemoglobin, white blood cell (WBC), neutrophil, lymphocyte, monocyte, and platelet counts, fibrinogen, blood urea nitrogen (BUN), creatinine, and C-reactive protein (CRP), were routinely measured at initial presentation in women with PPROM, immediately following diagnosis and before delivery. In the control group, the same laboratory parameters were obtained during routine outpatient visits at corresponding gestational ages.

For YKL-40 measurement, an additional 2 mL venous blood sample was obtained from each participant at enrollment. In the PPROM group, blood sampling was performed at the time of diagnosis and before the initiation of PPROM-related medical treatment, including antibiotics and antenatal corticosteroids, whereas control samples were obtained during the corresponding routine antenatal visit. Samples were centrifuged at 5000 rpm for 10 min, and the separated serum was stored at −80 °C until analysis. At the time of blood sampling, no participant had clinical chorioamnionitis and no participant was in active labor; clinical chorioamnionitis developed subsequently, during expectant management, in three women with PPROM.

Serum YKL-40 (chitinase-3-like protein 1 [CHI3L1]) concentrations were measured using a commercially available double-antibody sandwich enzyme-linked immunosorbent assay (ELISA) kit (Human Chitinase-3-like Protein 1 [YKL-40/CHI3L1] ELISA Kit; Cat. No. CK-bio-10841; Shanghai Coon Koon Biotech, Shanghai, China), according to the manufacturer’s instructions. In accordance with the assay protocol, 10 µL of serum was combined with 40 µL of sample diluent in each well, corresponding to a 5-fold sample dilution, whereas calibrators were applied undiluted. Concentrations interpolated from the standard curve were therefore multiplied by the manufacturer-specified dilution factor of 5, and all YKL-40 values reported in this manuscript are dilution-corrected. Calibrators of 0, 2, 4, 8, 16 and 32 ng/mL were assayed on the same plate and the standard curve was fitted using a four-parameter logistic model. Absorbance was measured at 450 nm using a calibrated microplate reader, and sample concentrations were interpolated from the fitted curve. The manufacturer-reported measurement range was 1–32 ng/mL, with a sensitivity of 0.1 ng/mL. The intra-assay and inter-assay coefficients of variation were <7% and <10%, respectively.

The manufacturer’s stated measurement range of 1–32 ng/mL and sensitivity of 0.1 ng/mL refer to the concentration in the assayed well, that is to the calibrator scale, because calibrators are applied undiluted. Taking the mandatory 5-fold sample dilution into account, the corresponding validated range for serum is 5–160 ng/mL and the corresponding functional sensitivity is 0.5 ng/mL. Dilution-corrected serum concentrations in this cohort ranged from 1.07 to 12.80 ng/mL. All 88 samples were above the functional sensitivity of the assay, and all sample optical densities fell between the zero calibrator and the 4 ng/mL calibrator, so that every value was interpolated within the fitted standard curve rather than extrapolated beyond it. However, 65 of 88 samples (73.9%), comprising all 44 controls and 21 of 44 PPROM cases, fell below the lower limit of the validated quantitative range once the dilution factor is taken into account. Concentrations in this cohort should therefore be regarded as detectable but not formally quantifiable against the manufacturer’s validated range.

### 2.6. Statistical Analysis

Statistical analyses were performed using IBM SPSS Statistics version 27.0 (IBM Corp., Armonk, NY, USA). The distribution of continuous variables was assessed using the Shapiro–Wilk test and visual inspection of histograms and Q–Q plots. Normally distributed continuous variables were presented as mean ± standard deviation (SD) and compared using the independent-samples *t*-test, whereas non-normally distributed variables were presented as median (interquartile range [IQR]) and compared using the Mann–Whitney U test. Categorical variables were expressed as numbers and percentages and compared using Pearson’s chi-square test or Fisher’s exact test, as appropriate. Correlations between YKL-40 levels and continuous variables were assessed using Pearson or Spearman correlation coefficients according to data distribution.

Multivariable Firth’s penalized logistic regression was used to evaluate factors independently associated with PPROM and, separately and within the PPROM cohort only, composite adverse neonatal outcome (CANO). For the PPROM model, variables with *p* < 0.20 in univariable analyses and those considered clinically relevant were included. Applying this rule, maternal age, hemoglobin, white blood cell count, neutrophil count, platelet count, fibrinogen, albumin, creatinine, C-reactive protein and YKL-40 met the threshold. Multicollinearity was assessed using variance inflation factors. Neutrophil count was strongly correlated with white blood cell count (r = 0.934), and when both were entered the variance inflation factors were 8.72 and 8.22; neutrophil count was therefore excluded and white blood cell count retained as the more general leukocyte index. After this exclusion, all variance inflation factors were below 1.6. The resulting model contains nine covariates for 44 events, corresponding to approximately five events per variable, and is therefore also reported as exploratory. For the CANO analysis, the model was prespecified rather than selected from the data and was restricted to variables that were available at the time of blood sampling, namely gestational age at PPROM diagnosis and maternal serum YKL-40. Gestational age at delivery was not entered into the primary CANO model, because it is known only after the biomarker measurement and lies on the causal pathway between PPROM severity, latency and neonatal condition; it was instead examined in a separate secondary model additionally adjusted for gestational age at delivery. This secondary model is an adjusted regression model and not a formal mediation analysis; direct and indirect effects were not estimated. Given the small number of non-events, both CANO models were limited to a maximum of three covariates and are reported as exploratory and hypothesis-generating. Firth’s penalized logistic regression was selected to reduce small-sample and separation-related bias. Results were reported as adjusted odds ratios (aORs) with 95% confidence intervals (CIs). With 13 neonates in the non-CANO group, the primary CANO model corresponds to approximately 6.5 and the secondary model to approximately 4.3 non-events per covariate, both below the conventional minimum of ten. The odds ratios and confidence intervals obtained from these models are therefore reported as imprecise estimates, and the absence of a statistically significant association is not interpreted as evidence that no association exists.

Adjusted odds ratios for YKL-40 are reported per 1 ng/mL increase on the dilution-corrected scale. Because observed serum creatinine spanned a narrow range, the corresponding aOR is reported per 0.1 mg/dL increase.

Receiver operating characteristic (ROC) curve analysis was performed as an exploratory analysis to assess the ability of maternal serum YKL-40 to discriminate between women with established PPROM and healthy controls. The area under the curve (AUC) was reported with 95% CIs. The optimal cut-off value was determined using the Youden index. Because YKL-40 was measured after PPROM diagnosis, ROC analysis was interpreted as case–control discrimination rather than prospective prediction. The cut-off derived in this analysis is presented as an exploratory finding only. The assay used is labeled by the manufacturer for research use and not for diagnostic application, and the cut-off has not been validated in an independent population; it is therefore not proposed as a clinically applicable threshold.

Because a substantial proportion of samples fell below the lower limit of the validated quantitative range, a prespecified sensitivity analysis was performed on the raw optical density values, which are independent of the calibration of the standard curve. Because the four-parameter logistic fit is a monotone transformation of optical density, rank-based comparisons and the area under the ROC curve are mathematically invariant to it. Missing data were handled using complete-case analysis without imputation. All statistical tests were two-sided, and *p* < 0.05 was considered statistically significant.

During the preparation of this manuscript, the author used Claude (Anthropic, San Francisco, CA, USA) for the purposes of language and translation editing, and for verification of the internal consistency of the reported statistics against the source dataset. The author reviewed and edited all content generated with the assistance of this tool and takes full responsibility for the content of this publication.

## 3. Results

**Figure 1:**
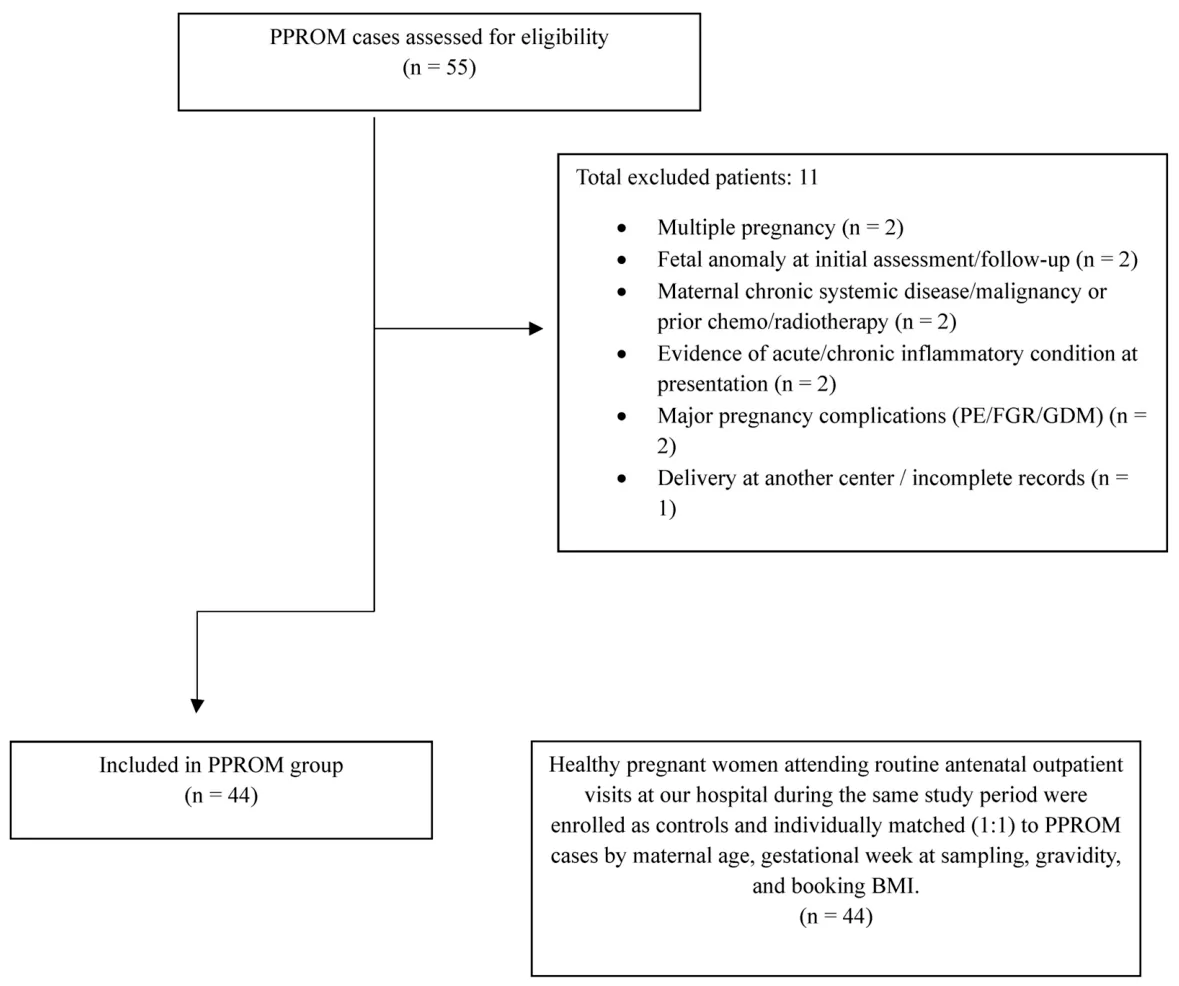
Flowchart of participant selection.

A total of 44 women with PPROM and 44 contemporaneously recruited healthy controls were included in the final analysis (Figure 1). The demographic, clinical, and laboratory characteristics of the groups are presented in Table 1. Maternal age, gravidity, parity, number of previous abortions, BMI, and gestational age at blood sampling were comparable between the groups (all *p* > 0.05). As expected, gestational age at delivery (32.17 ± 2.76 vs. 38.67 ± 1.06 weeks) and birth weight (1940 ± 771 vs. 3219 ± 361 g) were significantly lower in the PPROM group (both *p* < 0.001). Among laboratory parameters, platelet (*p* = 0.003) and fibrinogen (*p* = 0.007) levels were significantly higher, whereas albumin (*p* < 0.001) and creatinine (*p* = 0.003) levels were significantly lower in the PPROM group. Maternal serum YKL-40 concentrations were significantly higher in women with PPROM than in healthy controls (5.87 ± 2.64 vs. 2.44 ± 0.96 ng/mL, *p* < 0.001).

Obstetric and neonatal outcomes of the PPROM cohort are presented in Table 2. The median gestational age at delivery was 34.0 weeks (IQR, 30.9–34.0), and the mean birth weight was 1940 ± 771 g. NICU admission was required in 77.3% of neonates, while 45.5% required delivery-room resuscitation and 68.2% received oxygen support. Mechanical ventilation was required in 29.5% of neonates. Respiratory distress syndrome occurred in 15.9%, surfactant therapy was administered in 22.7%, and inotropic support was required in 29.5%. Neonatal sepsis occurred in 18.2%, while intraventricular hemorrhage and necrotizing enterocolitis were each observed in 2.3% of cases. Pulmonary hypoplasia occurred in 6.8%, and early neonatal death occurred in 11.4%; survival at discharge was 88.6%. Overall, CANO was observed in 31 of 44 neonates (70.5%).

Maternal, clinical, and laboratory characteristics according to CANO status within the PPROM cohort are presented in Table 3. Maternal age, BMI, and gestational age at PPROM diagnosis and blood sampling were comparable between the CANO and non-CANO groups (all *p* > 0.05). Gestational age at delivery (32.30 [29.80–34.00] vs. 34.00 [34.00–34.00] weeks, *p* = 0.002) and birth weight (1615 ± 558 vs. 2715 ± 657 g, *p* < 0.001) were significantly lower in the CANO group, whereas the latency period did not differ significantly (19.4 ± 15.7 vs. 28.1 ± 23.4 days, *p* = 0.158). No significant differences were observed for any laboratory parameter, including WBC count (*p* = 0.690), CRP (*p* = 0.758) and YKL-40 (*p* = 0.545).

Correlations between maternal serum YKL-40 levels and maternal, laboratory, and perinatal variables are presented in Table 4. No significant correlations were observed between YKL-40 levels and maternal age, BMI, WBC, neutrophil, lymphocyte, monocyte, or CRP levels in either the PPROM or control group (all *p* > 0.05). Within the PPROM cohort, YKL-40 was inversely correlated with gestational age at blood sampling (r = −0.304, *p* = 0.045), whereas no significant correlation was observed in the control group (r = 0.145, *p* = 0.348). Within the PPROM cohort, YKL-40 levels were also not significantly correlated with the latency period or 1- and 5-min Apgar scores (all *p* > 0.05).

Multivariable Firth penalized logistic regression analyses are presented in Table 5. In the PPROM model, maternal serum YKL-40 remained independently associated with PPROM status (aOR 5.61, 95% CI 2.07–15.23, *p* < 0.001 per 1 ng/mL increase), whereas the other included covariates were not significantly associated with PPROM. In the primary CANO model, which was restricted to the PPROM cohort and to variables available at the time of blood sampling, neither gestational age at PPROM diagnosis (aOR 0.844, 95% CI 0.679–1.050, *p* = 0.129) nor YKL-40 (aOR 1.041, 95% CI 0.789–1.374, *p* = 0.775) was significantly associated with CANO. In the secondary model additionally adjusted for gestational age at delivery, that variable was associated with CANO (aOR 0.295, 95% CI 0.093–0.933, *p* = 0.038), while YKL-40 remained non-significant (*p* = 0.565). These estimates are based on 31 neonates with and 13 without the composite outcome, corresponding to fewer than ten non-events per covariate; the width of the reported confidence intervals should be taken into account accordingly, and the non-significant results should not be read as excluding a clinically meaningful association.

**Figure 2:**
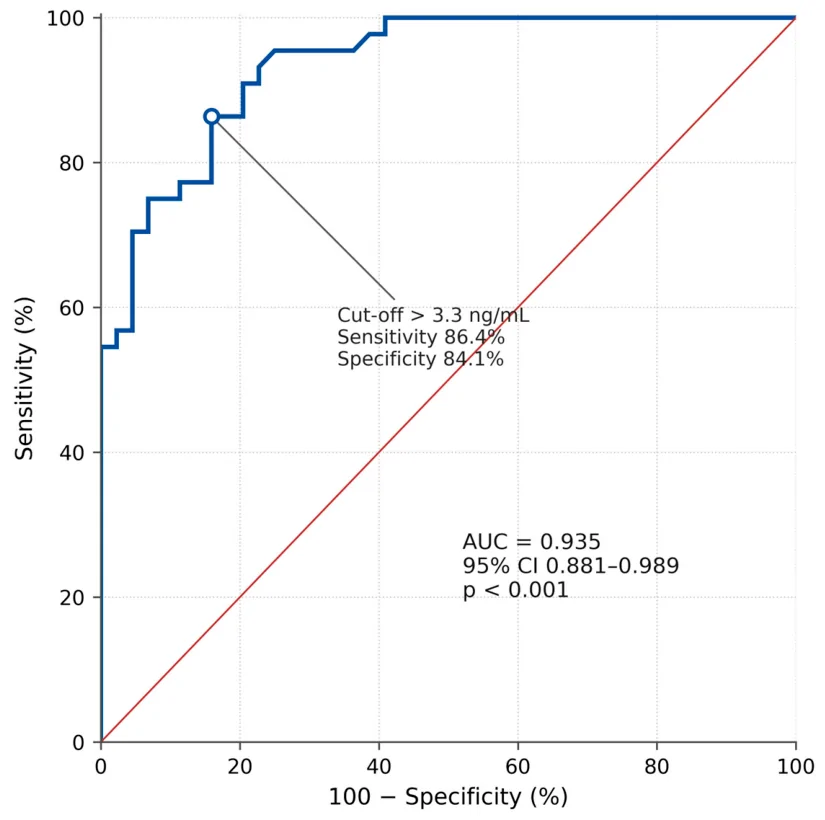
ROC curve for the discrimination of established PPROM cases from healthy controls using maternal serum YKL-40.

Exploratory ROC analysis demonstrated an AUC of 0.935 (95% CI 0.881–0.989, *p* < 0.001) for discriminating established PPROM cases from healthy controls (Figure 2). The Youden index identified an optimal YKL-40 cut-off of >3.3 ng/mL, yielding a sensitivity of 86.4% (95% CI 73.3–93.6%) and a specificity of 84.1% (95% CI 70.6–92.1%). Because YKL-40 was measured after PPROM diagnosis, these findings represent case–control discrimination rather than prospective prediction. In the prespecified sensitivity analysis using raw optical density values, the between-group difference remained highly significant (0.205 ± 0.051 vs. 0.133 ± 0.022, *p* < 0.001) and the area under the curve was identical (0.935), confirming that the discrimination observed does not depend on the calibration of the standard curve in its lowest segment.

## 4. Discussion

To the best of our knowledge, this is the first study to investigate maternal serum YKL-40 levels in pregnancies complicated by PPROM. Maternal YKL-40 levels were significantly higher in women with PPROM than in healthy pregnant controls and remained independently associated with PPROM after adjustment for potential confounders. In contrast, YKL-40 levels did not differ according to composite adverse neonatal outcome (CANO) status and were not independently associated with CANO within the PPROM cohort. Exploratory ROC analysis showed a high ability of YKL-40 to discriminate women with established PPROM from healthy controls. However, this finding should be interpreted in the context of the study design, as YKL-40 was measured after PPROM had already been diagnosed and the comparison involved women with overt PPROM and healthy pregnancies. Therefore, the observed discrimination does not demonstrate prospective predictive ability or establish diagnostic utility in clinical practice. The performance of YKL-40 in clinically relevant populations, such as women presenting with suspected PPROM, equivocal symptoms, or threatened preterm labor with intact membranes, remains to be determined.

The biomechanical integrity of the fetal membranes depends on tightly regulated extracellular matrix (ECM) homeostasis, including the balance between matrix metalloproteinases (MMPs) and their tissue inhibitors (TIMPs) [17]. In PPROM, infectious or sterile inflammatory activation can disrupt this balance, with proinflammatory cytokines such as IL-1β, IL-6, IL-8, and TNF-α promoting MMP activity and subsequent collagen degradation, thereby contributing to progressive weakening of the amnion and chorion [17,18,19]. This inflammatory cascade may also promote apoptosis of decidual and amniotic cells, further compromising fetal membrane integrity [20]. Elevated levels of proinflammatory cytokines, including IL-6 and IL-8, have also been associated with increased acute-phase reactants and systemic inflammatory markers in PPROM, further supporting the contribution of inflammatory activation to membrane rupture and its associated complications [21,22]. This inflammation-driven ECM remodeling provides a biologically plausible framework for our finding of elevated maternal YKL-40 levels in women with PPROM.

YKL-40 is involved in extracellular matrix remodeling through pathways including Akt/MMP-9 signaling, while its expression can be stimulated by proinflammatory cytokines such as IL-6 and IL-17 [14]. This dual involvement in inflammation and tissue remodeling provides further biological support for its potential association with PPROM. YKL-40 has also been investigated in several pregnancy-related disorders. Tepe et al. reported higher maternal serum YKL-40 levels in pregnancies complicated by placenta accreta spectrum [23], while Liu et al. demonstrated that YKL-40 promotes trophoblast proliferation and invasion, supporting its involvement in placental tissue remodeling [24]. Altered circulating YKL-40 levels have also been reported in other pregnancy complications, although its clinical significance across these conditions remains uncertain [25,26]. Taken together, these observations support the concept that elevated YKL-40 in PPROM may reflect inflammatory and extracellular matrix-remodeling activity rather than a disease-specific response.

The absolute YKL-40 concentrations observed in our cohort were lower than those reported in some previous obstetric series, in which cut-off values in the range of tens of ng/mL have been described. Circulating YKL-40 is known to vary substantially between commercial immunoassays because of differences in antibody pairs and calibrator material, and absolute concentrations from different platforms are therefore not directly comparable. This reinforces the point that the cut-off derived here is assay-specific and cannot be transferred to other assays or to clinical practice without independent analytical validation.

We also observed an inverse correlation between YKL-40 and gestational age at blood sampling within the PPROM cohort. Serum YKL-40 has previously been reported to vary across gestation, and this observation raises the possibility that gestational age contributes to the variability of YKL-40 in PPROM. Because gestational age at sampling was closely balanced between the groups by design, it is unlikely to explain the between-group difference we observed, but it should be taken into account in future studies with serial sampling.

The absence of an association between YKL-40 and the composite neonatal outcome should also be read against what is known to drive neonatal morbidity after preterm birth. Gestational age at delivery is a dominant determinant of early respiratory outcome, and the underlying obstetric condition contributes further risk beyond prematurity itself. In a recent cohort of pregnancies complicated by placenta praevia, each additional week of gestation was independently associated with substantially lower odds of respiratory resuscitation, non-invasive respiratory support and supplemental oxygen requirement, and histopathologically confirmed placenta accreta spectrum remained associated with respiratory morbidity after adjustment for gestational age [27]. In our cohort, gestational age at delivery and birth weight were the only variables that differed significantly between neonates with and without the composite outcome, and delivery was largely protocol-driven at 34 + 0 weeks. It is therefore plausible that neonatal morbidity in PPROM is determined principally by gestational age at delivery and by the obstetric context, leaving limited residual variance for a single maternal biomarker measured at the time of membrane rupture to explain.

This study has several strengths. The prospective enrollment of participants and collection of maternal blood samples at a standardized clinical time point, together with contemporaneous recruitment of healthy controls at comparable gestational ages, reduced potential variability related to sampling conditions. In addition, neonatal outcomes were systematically assessed, allowing evaluation of both the association of YKL-40 with established PPROM and its relationship with subsequent neonatal outcomes. Finally, the use of multivariable analyses enabled assessment of the association between YKL-40 and PPROM after adjustment for potential confounding factors.

This study also has several limitations. First, its single-center design and relatively small sample size may limit the generalizability of the findings. Second, the study design compared women with overt, established PPROM against healthy pregnant controls, and did not include the comparison groups that would be required to establish diagnostic utility, namely women presenting with suspected PPROM and intact membranes, women in preterm labor with intact membranes, or women delivering preterm for other indications. The discriminatory performance reported here therefore cannot be extrapolated to the clinical setting in which a diagnostic test would actually be used. A design capable of answering the diagnostic question would need to enroll, alongside confirmed cases, women presenting with clinically suspected but unconfirmed membrane rupture, women in preterm labour with intact membranes, and women delivering preterm for other indications, and would need to be sized a priori for discrimination among these groups rather than for a two-group comparison of concentrations. Because the comparator group here consisted of uncomplicated pregnancies, the discrimination observed is best regarded as an upper bound, and performance against clinically similar conditions would be expected to be lower. Third, YKL-40 was measured on a single commercial research-use assay, in single wells rather than in duplicate, and in a single analytical run; no independent method comparison was performed, and the reported concentrations and cut-off are consequently assay-specific. A related and more important analytical limitation is that, once the mandatory 5-fold sample dilution is taken into account, the validated quantitative range of the assay corresponds to 5–160 ng/mL of serum, whereas 65 of 88 samples (73.9%) fell below this range. The reported concentrations are therefore detectable but not formally quantifiable, and the absolute values and the ROC-derived cut-off must be regarded as provisional. The prespecified sensitivity analysis on raw optical density gave an identical area under the curve, so the discriminatory finding itself does not depend on the calibration of the standard curve; nevertheless, an assay with a lower quantitative range, or measurement of undiluted serum, would be required before any threshold could be proposed for use. Because no participant had clinical chorioamnionitis or was in active labor at the time of blood sampling, the influence of intra-amniotic infection and of established labor on circulating YKL-40 could not be examined within this cohort; our findings therefore describe YKL-40 at the time of membrane rupture rather than in the presence of overt intra-amniotic infection. In addition, YKL-40 was measured at a single time point, precluding evaluation of temporal changes during the course of PPROM. The composite adverse neonatal outcome (CANO) included events of differing clinical severity and was analyzed as an unweighted binary endpoint; although individual components were reported separately, this heterogeneity should be considered when interpreting the composite outcome, and the number of individual severe events, such as intraventricular hemorrhage, necrotizing enterocolitis and death, was too small to permit separate multivariable analysis of a more homogeneous endpoint. Furthermore, only 13 women were included in the non-CANO group, substantially limiting the robustness of the multivariable analysis despite the use of Firth’s penalized logistic regression and increasing the possibility of a Type II error; the CANO models should therefore be regarded as exploratory. No a priori sample-size calculation was performed for the ROC or CANO analyses, and with 13 non-events for two to three covariates the events-per-variable ratio lies below accepted recommendations; a clinically meaningful association between YKL-40 and neonatal outcome could therefore have been missed. An additional consideration is that, under the institutional protocol, delivery was recommended at 34 + 0 weeks, so gestational age at delivery in this cohort was partly determined by management policy; associations involving this variable should be interpreted accordingly. Finally, controls were not systematically reassessed after enrollment for the subsequent development of pregnancy complications; therefore, later-onset complications among controls cannot be completely excluded. These limitations emphasize the need for larger, multicenter prospective studies with serial biomarker measurements, structured assessment of potential confounders, and clinically well-defined neonatal endpoints.

## 5. Conclusions

Maternal serum YKL-40 levels were significantly elevated in women with established PPROM compared with healthy pregnant controls, supporting a potential association with the inflammatory and extracellular matrix-remodeling processes involved in membrane rupture. However, YKL-40 was not associated with subsequent composite adverse neonatal outcomes in this cohort. Because YKL-40 was measured after PPROM diagnosis, the present findings do not establish its ability to predict PPROM before clinical onset or its diagnostic utility in symptomatic populations. Further multicenter prospective studies using clinically relevant comparison groups and independently validated assays are needed to determine whether YKL-40 has potential clinical value in PPROM. Such studies should include women with clinically suspected but unconfirmed membrane rupture, women in preterm labour with intact membranes and women delivering preterm for other indications, and should be sized a priori for diagnostic discrimination and for the prediction of neonatal outcome rather than for the comparison of biomarker concentrations alone.

## Figures and Tables

**Table 1:** Comparison of demographic, clinical, and laboratory characteristics between PPROM and control groups.

Variables	PPROM Group (*n* = 44)	Control Group (*n* = 44)	*p*-Value
Maternal Age (years)	29.48 ± 6.17	27.89 ± 5.13	0.192 a
Gravidity (*n*)	2.00 (1.00–3.00)	2.50 (1.00–3.00)	0.488 b
Parity (*n*)	1.00 (0.00–2.00)	1.00 (0.00–2.00)	0.570 b
Abortus (*n*)	0.00 (0.00–1.00)	0.00 (0.00–0.00)	0.376 b
BMI (kg/m^2^)	28.00 (25.75–31.00)	29.00 (26.00–31.25)	0.257 b
Gestational age at blood sampling (week)	29.28 ± 3.32	28.69 ± 3.37	0.408 a
Gestational age at delivery (weeks)	32.17 ± 2.76	38.67 ± 1.06	<0.001 a
Birth weight (g)	1940.36 ± 771.38	3218.86 ± 360.82	<0.001 a
Oligohydramnios	4 (9.1%)	-	0.116 c
Development of chorioamnionitis	3 (6.8%)	1 (2.3%)	0.616 c
Placental abruption	2 (4.5%)	-	0.494 c
Laboratory parameters			
Hemoglobin (g/dL)	11.19 ± 1.47	11.57 ± 1.10	0.179 a
WBC (10^3^/µL)	11.52 (10.23–13.46)	10.81 (9.72–11.55)	0.076 b
Neutrophil count (10^3^/µL)	8.36 (7.44–10.74)	7.75 (7.05–8.60)	0.059 b
Lymphocyte count (10^3^/µL)	1.99 ± 0.74	1.96 ± 0.49	0.851 a
Monocyte count (10^3^/µL)	0.72 ± 0.30	0.73 ± 0.15	0.715 a
Platelet (10^3^/µL)	273.82 ± 71.86	233.41 ± 49.54	0.003 a
Fibrinogen (mg/dL)	483.00 (412.75–575.75)	452.50 (346.75–506.00)	0.007 b
Albumin (g/L)	36.30 ± 2.46	38.77 ± 2.86	<0.001 a
BUN (mg/dL)	15.11 ± 5.87	16.22 ± 3.50	0.282 a
Creatinine (mg/dL)	0.49 ± 0.11	0.57 ± 0.14	0.003 a
CRP (mg/L)	6.23 (3.21–12.64)	4.90 (3.30–7.90)	0.182 b
YKL-40 (ng/mL)	5.87 ± 2.64	2.44 ± 0.96	<0.001 a

a Independent-samples *t*-test. Data are presented as mean ± standard deviation. b Mann–Whitney U test. Data are presented as median (interquartile range). c Fisher’s exact test. Data are presented as *n* (%). Abbreviations: PPROM, preterm prelabor rupture of membranes; BMI, body mass index; WBC, white blood cell count; BUN, blood urea nitrogen; CRP, C-reactive protein; YKL-40, chitinase-3-like protein 1.

**Table 2:** Obstetric and neonatal outcomes of the PPROM cohort (*n* = 44).

Variables	Value
Gestational age at delivery (weeks), median (IQR)	34.0 (30.9–34.0)
Birth weight (g), mean ± SD	1940 ± 771
Fetal distress, *n* (%)	6 (13.6%)
Apgar score at 1st minute, median (IQR)	7 (6–8)
Apgar score at 5th minute, median (IQR)	8 (7–9)
Apgar score ≤ 7 at 5 min, *n* (%)	17 (38.6%)
Umbilical cord arterial pH, median (IQR) [*n* = 35]	7.29 (7.20–7.36)
NICU admission, *n* (%)	34 (77.3%)
Duration of NICU stay (days), median (IQR)	8 (1–26)
Neonatal acidosis, *n* (%)	8 (18.2%)
Need for resuscitation, *n* (%)	20 (45.5%)
Need for oxygen support, *n* (%)	30 (68.2%)
Duration of oxygen support (days), median (IQR)	3 (0–10)
Mechanical ventilation, *n* (%)	13 (29.5%)
Duration of mechanical ventilation (days), median (IQR)	0 (0–1)
Development of RDS, *n* (%)	7 (15.9%)
Need for surfactant therapy, *n* (%)	10 (22.7%)
Need for inotropic support, *n* (%)	13 (29.5%)
Sepsis, *n* (%)	8 (18.2%)
Intraventricular hemorrhage, *n* (%)	1 (2.3%)
Necrotizing enterocolitis, *n* (%)	1 (2.3%)
Pulmonary hypoplasia, *n* (%)	3 (6.8%)
Early neonatal death, *n* (%)	5 (11.4%)
Survival at discharge, *n* (%)	39 (88.6%)
CANO, *n* (%)	31 (70.5%)

Abbreviations: PPROM, preterm prelabor rupture of membranes; CANO, composite adverse neonatal outcome; NICU, neonatal intensive care unit; RDS, respiratory distress syndrome.

**Table 3:** Comparison of maternal, clinical, and laboratory characteristics between CANO and non-CANO groups in pregnancies complicated by PPROM.

Variables	CANO (*n* = 31)	Non-CANO (*n* = 13)	*p*-Value
Maternal Age (years)	29.77 ± 6.30	28.77 ± 6.03	0.627 a
BMI (kg/m^2^)	28.45 ± 4.48	27.69 ± 4.63	0.614 a
Gestational age at PPROM and blood sampling (weeks)	28.66 ± 3.45	30.71 ± 2.82	0.066 a
Latency period (days)	19.42 ± 15.67	28.08 ± 23.37	0.158 a
Gestational age at delivery (weeks)	32.30 (29.80–34.00)	34.00 (34.00–34.00)	0.002 c
Birth Weight (g)	1615.35 ± 557.53	2715.38 ± 656.87	<0.001 a
Oligohydramnios	4 (12.9%)	-	0.302 b
Development of chorioamnionitis	3 (9.7%)	-	0.544 b
Placental abruption	2 (6.5%)	-	1.000 b
Laboratory parameters			
Hemoglobin (g/dL)	11.35 ± 1.50	10.82 ± 1.38	0.277 a
WBC (10^3^/µL)	11.71 (10.41–13.12)	11.00 (9.46–15.81)	0.690 c
Neutrophil count (10^3^/µL)	8.35 (7.50–10.49)	8.41 (7.44–12.62)	0.797 c
Lymphocyte count (10^3^/µL)	1.93 ± 0.67	2.12 ± 0.90	0.434 a
Monocyte count (10^3^/µL)	0.73 ± 0.30	0.67 ± 0.33	0.565 a
Platelet (10^3^/µL)	266.68 ± 69.66	290.85 ± 77.00	0.314 a
Fibrinogen (mg/dL)	509.32 ± 115.17	486.15 ± 79.63	0.513 a
Albumin (g/L)	36.54 ± 2.68	35.75 ± 1.82	0.338 a
BUN (mg/dL)	15.46 ± 6.16	14.27 ± 5.24	0.547 a
Creatinine (mg/dL)	0.48 ± 0.10	0.51 ± 0.14	0.393 a
CRP (mg/L)	6.45 (3.19–11.85)	6.01 (3.84–18.59)	0.758 c
YKL-40 (ng/mL)	5.31 (3.92–7.75)	5.18 (3.77–6.36)	0.545 c

a Independent-samples *t*-test. Data are presented as mean ± standard deviation. b Fisher’s exact test. Data are presented as *n* (%). c Mann–Whitney U test. Data are presented as median (interquartile range). Abbreviations: PPROM, preterm prelabor rupture of membranes; CANO, composite adverse neonatal outcome; BMI, body mass index; WBC, white blood cell count; BUN, blood urea nitrogen; CRP, C-reactive protein; YKL-40, chitinase-3-like protein 1.

**Table 4:** Correlation between maternal, perinatal, and laboratory variables and YKL-40 levels in the PPROM and control cohorts.

Variables	PPROM (*n* = 44)		Control (*n* = 44)	
	r	*p*-Value	r	*p*-Value
Maternal Age (years)	0.032	0.838 b	0.047	0.761 b
BMI (kg/m^2^)	0.166	0.282 b	−0.195	0.204 b
Gestational age at blood sampling (week)	−0.304	0.045 b	0.145	0.348 b
WBC (10^3^/µL)	−0.157	0.309 a	0.112	0.471 a
Neutrophil count (10^3^/µL)	−0.255	0.095 a	0.120	0.436 a
Lymphocyte count (10^3^/µL)	−0.234	0.126 b	0.097	0.533 b
Monocyte count (10^3^/µL)	−0.014	0.926 b	0.063	0.682 b
CRP (mg/L)	−0.087	0.572 a	0.193	0.209 a
Latency period (days)	−0.093	0.546 b	-	-
Apgar score at 1st minute	−0.146	0.345 a	0.020	0.896 a
Apgar score at 5th minute	−0.249	0.103 a	0.032	0.835 a
CANO status	0.058	0.710 a	-	-
Survival at discharge	−0.115	0.458 a	-	-

a Spearman correlation coefficient. b Pearson correlation coefficient. Abbreviations: PPROM, preterm prelabor rupture of membranes; CANO, composite adverse neonatal outcome; BMI, body mass index; WBC, white blood cell count; CRP, C-reactive protein.

**Table 5:** Multivariable Firth’s penalized logistic regression analyses.

Variables	aOR	95% CI	*p*-Value
Model 1. Factors associated with PPROM (44 PPROM vs. 44 controls)			
Maternal age (years)	1.020	0.884–1.178	0.783
Hemoglobin (g/dL)	0.961	0.567–1.630	0.883
WBC (10^3^/µL)	1.107	0.889–1.377	0.365
Platelet (10^3^/µL)	1.010	0.997–1.023	0.131
Fibrinogen (mg/dL)	1.009	0.997–1.021	0.139
Albumin (g/L)	0.966	0.719–1.298	0.820
Creatinine (per 0.1 mg/dL)	0.683	0.361–1.292	0.241
CRP (mg/L)	0.956	0.824–1.110	0.558
YKL-40 (per 1 ng/mL)	5.609	2.065–15.234	<0.001
Model 2. Factors associated with CANO within the PPROM cohort—primary model (variables available at blood sampling; 31 events/13 non-events)			
Gestational age at PPROM/sampling (weeks)	0.844	0.679–1.050	0.129
YKL-40 (per 1 ng/mL)	1.041	0.789–1.374	0.775
Model 3. Secondary model—additionally adjusted for gestational age at delivery			
Gestational age at PPROM/sampling (weeks)	1.101	0.825–1.469	0.512
Gestational age at delivery (weeks)	0.295	0.093–0.933	0.038
YKL-40 (per 1 ng/mL)	1.099	0.797–1.515	0.565

Adjusted odds ratios for YKL-40 are expressed per 1 ng/mL increase on the dilution-corrected scale, and for creatinine per 0.1 mg/dL increase. Model 1 includes all variables with *p* < 0.20 in univariable analysis except neutrophil count, which was excluded because of collinearity with white blood cell count (r = 0.934; variance inflation factors 8.72 and 8.22 when both were entered). After exclusion, all variance inflation factors were below 1.6. Model 1 contains nine covariates for 44 events (approximately five events per variable). Model 3 is an adjusted regression model, not a formal mediation analysis. Models 2 and 3 are restricted to the PPROM cohort and are exploratory. They contain 13 non-events for two and three covariates respectively, corresponding to approximately 6.5 and 4.3 non-events per variable. Abbreviations: PPROM, preterm prelabor rupture of membranes; CANO, composite adverse neonatal outcome; aOR, adjusted odds ratio; CI, confidence interval; WBC, white blood cell count; CRP, C-reactive protein; YKL-40, chitinase-3-like protein 1.

## Data Availability

The data presented in this study are available on request from the corresponding author. The data are not publicly available because they contain information that could compromise the privacy of the participants.

## Supplementary Materials

The following supporting information can be downloaded at: https://www.mdpi.com/article/10.3390/diagnostics16172845/s1, Table S1: Definitions and frequencies of the individual components of the composite adverse neonatal outcome (CANO) in the PPROM cohort.

## Abbreviations

PPROM: preterm prelabor rupture of membranes; CANO: composite adverse neonatal outcome; CHI3L1: chitinase-3-like protein 1; ELISA: enzyme-linked immunosorbent assay; ROC: receiver operating characteristic; AUC: area under the curve; CI: confidence interval; aOR: adjusted odds ratio; BMI: body mass index; WBC: white blood cell; CRP: C-reactive protein; BUN: blood urea nitrogen; NST: non-stress test; ACOG: American College of Obstetricians and Gynecologists; NICU: neonatal intensive care unit; RDS: respiratory distress syndrome; IVH: intraventricular hemorrhage; NEC: necrotizing enterocolitis; MMP: matrix metalloproteinase; TIMP: tissue inhibitor of metalloproteinase; ECM: extracellular matrix; SGA: small for gestational age; GDM: gestational diabetes mellitus; ICP: intrahepatic cholestasis of pregnancy; PAS: placenta accreta spectrum; SD: standard deviation; IQR: interquartile range; CV: coefficient of variation; PAPP-A: pregnancy-associated plasma protein-A.
