# Supplementary material for: The Significance of YKL-40, a Marker Involved in Extracellular Matrix Remodeling, in Preterm Prelabor Rupture of Membranes: A Prospective Observational Study
Source: Diagnostics (Basel). 2026 Sep 4;16(17):2845. doi: 10.3390/diagnostics16172845 (PMC13564601; doi:10.3390/diagnostics16172845)
Supplement: Supplementary file 1 [file diagnostics-16-02845-s001.zip › diagnostics-4527455-supplementary.pdf]

Supplementary Table S1. Definitions and frequencies of the individual components of the composite adverse neonatal outcome (CANO) in the PPROM cohort

Component	Definition applied in this study	Source of data	n (%) N = 44
Neonatal metabolic acidosis	Umbilical or neonatal arterial pH <7.20 and/or base deficit >10 mmol/L and/or bicarbonate <15 mmol/L, as documented in the neonatal record.	Neonatal blood gas record	8 (18.2)
Need for delivery-room resuscitation	Resuscitation performed in the delivery room, beyond routine care at birth, comprising any of the following: positive-pressure ventilation by mask, endotracheal intubation, chest compressions, or administration of adrenaline. Recorded prospectively by the attending neonatology team on the standardized delivery-room resuscitation form. Continuous positive airway pressure applied alone, without any of the above, was not counted as resuscitation.	Delivery-room resuscitation record	20 (45.5)
Respiratory distress syndrome	Clinical respiratory distress with compatible chest radiographic findings, diagnosed by the attending neonatology team. Recorded as a binary outcome without severity staging.	Neonatal chart and chest radiograph	7 (15.9)
Surfactant requirement	Administration of at least one dose of exogenous surfactant.	Neonatal treatment record	10 (22.7)
Mechanical ventilation	Any duration of invasive endotracheal ventilation during the neonatal admission.	Neonatal chart	13 (29.5)
Prolonged oxygen support	Requirement for supplemental oxygen extending beyond immediate delivery-room resuscitation, for any duration.	Neonatal chart	30 (68.2)
Inotropic support	Administration of any inotropic or vasoactive agent for circulatory support.	Neonatal treatment record	13 (29.5)
Neonatal sepsis	Both culture-proven and clinically diagnosed neonatal sepsis were counted. Culture-proven sepsis required a positive blood culture together with compatible clinical signs. Clinical sepsis required compatible clinical signs (temperature instability, respiratory deterioration, feeding intolerance, lethargy or hemodynamic compromise) together with supporting laboratory findings (elevated C-reactive protein and/or an abnormal complete blood count) and the initiation of a full course of antibiotic therapy by the attending neonatology team, in the absence of a positive culture.	Neonatal chart and laboratory record	8 (18.2)
Intraventricular hemorrhage	Intraventricular hemorrhage of any grade on cranial ultrasound. Recorded as a binary outcome without grading.	Cranial ultrasound report	1 (2.3)
Necrotizing enterocolitis	Necrotizing enterocolitis diagnosed by the attending neonatology team. Recorded as a binary outcome without staging.	Neonatal chart	1 (2.3)
Pulmonary hypoplasia	Pulmonary hypoplasia diagnosed by the attending neonatology team on the basis of clinical and radiological findings in the context of prolonged oligohydramnios.	Neonatal chart	3 (6.8)
Early neonatal death	Death occurring within the first 7 completed days of life.	Hospital mortality record	5 (11.4)
**CANO (at least one component present)**			**31 (70.5)**

Neonatal intensive care unit (NICU) admission was not a component of CANO. Neonates admitted to the NICU for observation of prematurity alone, without any of the components listed above, were classified as not having CANO; three neonates fell into this category. NICU admission is reported separately in Table 2.

Distribution of the number of components per neonate

Number of CANO components	Neonates, n	%
0 (no CANO)	13	29.5
1	12	27.3

Number of CANO components	Neonates, n	%
2	4	9.1
3	1	2.3
4	1	2.3
5	4	9.1
6	1	2.3
7	4	9.1
8	1	2.3
9	1	2.3
10	1	2.3
11	1	2.3

Twelve of the 31 neonates meeting CANO did so through a single component, most often prolonged oxygen support, whereas 14 neonates had five or more components. This distribution illustrates the clinical heterogeneity of the composite and supports its interpretation as an exploratory endpoint.

Severe ("hard") neonatal outcomes

A more homogeneous sub-composite restricted to severe outcomes (intraventricular hemorrhage, necrotizing enterocolitis, pulmonary hypoplasia, or early neonatal death) occurred in 6 of 44 neonates (13.6%). Because of the small number of events, this sub-composite is reported descriptively only and was not entered into multivariable analysis.

Abbreviations: CANO, composite adverse neonatal outcome; PPROM, preterm prelabor rupture of membranes; NICU, neonatal intensive care unit.
